# Role of pericyte‐derived SENP1 in neuronal injury after brain ischemia

**DOI:** 10.1111/cns.13398

**Published:** 2020-06-04

**Authors:** Meiling Sun, Xiang Chen, Yi‐Xuan Yin, Yinping Gao, Li Zhang, Boqian Chen, Yin Ji, Kohji Fukunaga, Feng Han, Ying‐Mei Lu

**Affiliations:** ^1^ Key Laboratory of Cardiovascular & Cerebrovascular Medicine School of Pharmacy Nanjing Medical University Nanjing China; ^2^ Department of Physiology Nanjing Medical University Nanjing China; ^3^ Research Laboratory for Biomedical Optics and Molecular Imaging Shenzhen Institutes of Advanced Technology Chinese Academy of Sciences Shenzhen China; ^4^ School of Medicine Zhejiang University City College Hangzhou China; ^5^ Department of Geriatrics Nanjing Brain Hospital affiliated to Nanjing Medical University Nanjing China; ^6^ The State Key Laboratory of Translational Medicine and Innovative Drug Development Simcere Pharmaceutical Group Nanjing China; ^7^ Department of Pharmacology Graduate School of Pharmaceutical Sciences Tohoku University Sendai Japan

**Keywords:** apoptosis, brain ischemia, pericytes, SENP1, SUMOylation

## Abstract

**Aims:**

SUMOylation is a posttranslational modification related to multiple human diseases. SUMOylation can be reversed by classes of proteases known as the sentrin/SUMO‐specific proteases (SENPs). In the present study, we investigate the potential role of SENP1 in pericytes in the brain ischemia.

**Methods:**

Pericyte‐specific deletion of *senp1* mice (*Cspg4‐Cre; senp1^f/f^*) were used for brain function and neuronal damage evaluation following brain ischemia. The cerebral blood vessels of diameter, velocity, and flux were performed in living mice by two‐photon laser scanning microscopy (TPLSM). Biochemical analysis and immunohistochemistry methods were used to address the role and mechanism of pericyte‐specific SENP1 in the pathological process of brain ischemia. A coculture model of HBVPs and HBMECs mimicked the BBB in vitro and was used to evaluate BBB integrity after glucose deprivation.

**Results:**

Our results showed that *senp1*‐specific deletion in pericytes did not affect the motor function and cognitive function of mice. However, the pericyte‐specific deletion of *senp1* aggravated the infarct size and motor deficit following focal brain ischemia. Consistently, the TPLSM data demonstrated that SENP1 deletion in pericytes accelerated thrombosis formation in brain microvessels. We also found that pericyte‐specific deletion of *senp1* exaggerated the neuronal damage significantly following brain ischemia in mice. Moreover, SENP1 knockdown in pericytes could activate the apoptosis signaling and disrupt the barrier integrity in vitro coculture model.

**Conclusions:**

Our findings revealed that targeting SENP1 in pericytes may represent a novel therapeutic strategy for neurovascular protection in stroke.

## 1 INTRODUCTION

Stroke is the major cause of acquired adult disability and leading death worldwide.^1^, ^2^ Approximately 80% of stroke are ischemic stroke, resulting from the thromboembolic occlusion of a blood vessel. The pathophysiology of stroke is complex and involves numerous cell types, including neurons, glia, endothelial cells (ECs), pericytes, and so on.^3^, ^4^ Tissue‐type plasminogen activator (tPA) therapeutic strategy was restricted to patients receiving treatment within 4.5 hours from stroke onset, which only benefit for about 10% patients.^5^ Therefore, in order to find effective treatments in stroke, we need to identify novel mechanism‐based targets.^6^, ^7^

Neurons, pericytes, vascular ECs, astrocytes, and microglia compose the neurovascular unit (NVU).^8^ Pericytes are located in the NVU between astrocytes and endothelial cells, embedded in the basement membrane of blood microvessels.^9^ Pericytes could integrate and process signals from their neighboring cells, which are critical for central nervous system (CNS) functions, including regulation of the blood‐brain barrier (BBB) permeability, angiogenesis, capillary hemodynamic responses, and so on.^9^ Ischemic stroke destroyed the NVU rapidly, leading to pericytes loss, BBB breakdown, microglial activation, and finally neuronal death.^10^, ^11^ Evidences indicated that pericytes could damage BBB and control vascular constriction around infarction periphery, contributing to the process of ischemia.^12^ However, the molecular basis of the mechanisms in pericytes in brain ischemia is poorly understood. New insights into the mechanisms of pericytes might protect NVU from ischemia and result in potential therapeutic strategies for stroke.

Apart from phosphorylation and ubiquitination, SUMOylation is a posttranslational modification, characterized by adding or detaching small ubiquitin‐like modifier (SUMO) proteins to lysine residues on target proteins.^13^, ^14^ SUMOylation, a dynamic process, is mediated by the activating (E1), conjugating (E2), and ligating (E3) enzymes and has emerged as an important regulatory mechanism for protein localization and function.^14^, ^15^, ^16^, ^17^, ^18^, ^19^ The deconjugation of SUMOylation is mediated by sentrin/SUMO‐specific proteases (SENPs).^20^ SENP1, a member of the SENP family, could deconjugate a large number of SUMOylated proteins, such as HIPK and HIF‐1α.^20^, ^21^ SENP1 also involved in processing the precursor SUMO to generate their mature form.^22^ SENP1 has been implicated in the development of ischemia, such as the increasing expression of SENP1 in neurons in response to brain ischemia.^23^ Moreover, SENP1 overexpression could rescue cell death in ischemia/reperfusion (I/R) injury, indicating the protective role of SENP1 in neurons.^23^ However, little is known about SENP1 in pericytes in cerebral ischemia.

In this study, we specifically deleted SEPN1 in pericytes in mice to generate *Cspg4‐Cre; senp1^f/f^* mice. Then, we detected the effects of *senp1* deletion on cerebral ischemic damage in mice. Our results revealed a protective role of SENP1 in pericytes in cerebral ischemia in mice, which could become a new therapeutic strategy for brain ischemic stroke. Moreover, our study indicated pericytes as the potential targets for restoring NVU function, and consequently rescuing neuronal function in stroke.

## 2 MATERIALS AND METHODS

### 2.1 Transcriptional analysis of SUMOylation protein

Differentially expression genes(DEGs) were selected by false discovery rate (FDR) value <0.05 from three datasets including GSE39866,^24^ GSE52564,^25^ and GSE36010.^26^ And we searched the genes related to SUMOylation from the Human Protein Atlas. The bioinformatics analysis was mainly performed with R software (version 3.6.1, Bell Laboratories). Venn diagram^27^ was used to generate Venn plot. We used the R package pheatmap with scale function to visualize the expression values with scale function.^28^

### 2.2 Cell culture and siRNA interference

Human brain vascular pericytes (HBVPs) were purchased from ScienCell (#1200) and maintained in Dulbecco's modified Eagle's medium (DMEM, Gibco) supplemented with 10% fetal bovine serum (FBS, Gibco) and 1% penicillin/streptomycin at 37°C in 5% CO_2_‐humidified incubator. After reaching 80%‐90% confluence, the cells were passaged with trypsin (0.25%)‐EDTA (0.02%) in PBS at a split ratio of 1:5. The media were changed every 2 days.^29^

When reaching 60%‐70% confluence, the HBVPs were transfected with 10 μM Senp1 siRNA using Lipofectamine^®^ RNAiMAX Reagent (13778, Invitrogen) for 48 h as described in the manual guide. Then, the cells were cultured with glucose‐free Hanks' Balanced Salt Solution (HBSS: 116 mM NaCl, 5.4 mM KCl, 0.8 mM MgSO_4_, 1.0 mM NaH_2_PO_4_, 1.8 mM CaCl_2_, and 26 mM NaHCO_3_, pH 7.3) for another 6 h. Thereafter, the cells were captured or used for Western blotting assay and immunofluorescence assay.

### 2.3 Animals

Mice were housed under a 12/12 hours light/dark cycle at a constant temperature of 22 ± 1°C with 40%‐60% humidity provided access to standard food and water. Pericyte‐specific deletion of *senp1* (*Cspg4‐Cre; senp1^f/f^*) was generated by crossed transgenic *Cspg4‐Cre* mice (Jackson Labs, Stock No. 008533) with mice carrying a loxP‐flanked *senp1* gene (C57BL/6 background).^30^ *senp1^f/f^* mice were used as controls in this study. Experiments mice were cared for in accordance with National Institutes of Health guidelines, and procedures were approved by the Zhejiang University and Nanjing Medical University Animal Committee in China.

### 2.4 Photochemically induced ischemic stroke in mice

The photochemically induced thrombosis (PIT) model was prepared by Rose Bengal (Sigma‐Aldrich) injection in 12‐week‐old mice as described previously.^31^ Mice were anesthetized by chloral hydrate (400 mg/kg, i.p.), fixed on stereotaxic apparatus and exposed the skull. Rose Bengal was administered to mice at 100 mg/kg in saline (i.p.). 5 minutes later, skull was exposed in LED light, and illuminated square area was 1 mm^2^ at around 2 mm postbregma and 1.5 mm lateral for 20 minutes. After 24 hours, mice were sacrificed for further experiments.

### 2.5 TTC staining

Twenty‐four hours after the induction of PIT ischemia, the brains were removed and sectioned coronally into 2‐mm‐thick slices using blade and a metallic brain matrix. Brain slices were immersed in 1% triphenyltetrazolium chloride (TTC) solution in normal saline at 37°C for 10 minutes and shook every 3 minutes.^32^ Brain slices were photographed using a scanner, and the infarct volume, which was not stained with TTC, was measured by ImageJ.

### 2.6 Rotarod test

Prior to the start of testing, mice were trained for 10 minutes per day for two consecutive days at 10 rpm. Mice were put back on the rotarod if drop out. The equipment was cleaned by 75% ethyl alcohol between two group tests. In the period of the testing experiment, mice were tested at 30 rpm, and the time that mice drop out was recorded. If mice remain more than 10 minutes on rotarod, we record 10 minutes and stop test.^33^ On the next day, the mice were subjected to brain ischemia and tested again 24 hours after ischemia.

### 2.7 Y‐maze test

Y‐maze test was performed as described previously to examine spatial working memory.^34^ A Y‐maze device with three identical Plexiglas arms (31 cm × 7 cm × 14 cm, 120° apart) was placed at the center of a room under dim lighting conditions. The walls of each arm had a distinct design to provide visual cues. Y‐maze testing was carried out 3 minutes each group. At the beginning of the test, each mouse was placed at the end of one arm facing the center. Sessions were recorded by video, and arm entries were scored by a trained observer, blind to treatment group. The total number of arms entering during the sessions was recorded as locomotor activity of mice. The percentage of spontaneous alterations was calculated as the ratio, defined as consecutive entries into a new arm before returning to the two visited arms previously. Accurate rate was calculated as: Accurate rate=Number of successful alteration / (total arms entry ‐ 2).

### 2.8 Preparation of mice for Two‐Photon laser scanning microscopy (TPLSM) in vivo

Mice were anesthetized with chloral hydrate and prepared for in vivo imaging. A skull‐thin was generated by stereotaxic coordinates (2 mm in diameter and 2 mm later bregma, 1.5 mm lateral) to observe vascular thrombosis 3, 6, and 24 hours after local ischemia. The custom‐made metal frame (1 cm diameter) was used to fix. The cerebral blood vessels of diameter, velocity, and flux of mice were imaged through a craniotomy window, which centered at stereotaxic coordinates 2 mm caudal to bregma and 1.5 mm lateral to the bregma in vivo.^35^, ^36^ After removal of the dura, the 1‐cm‐diameter metal frame with a removable 4‐mm‐glass lid was glued to the skull. The space between the exposed brain surface and the cover glass was filled with saline.

### 2.9 TPLSM imaging and analysis

A two‐photon confocal microscope (Olympus, BX61W1‐FV1000), equipped with a femtosecond Ti:Sa laser excitation source and Spectra‐Physics MaiTai HP DeepSee, was used to acquire a stacked or single focal plane two‐photon image. A long working‐distance (2 mm) water‐immersion objective (× 25, NA 1.05) was used to measure blood flow and vascular thrombosis in mouse brain cortex. Intravenous injection of Texas Red Dextran solution (70 kD, Sigma‐Aldrich) was used for labeling blood plasma in vivo blood flow test. The imaging was obtained by XYT stack and XYZ stack. The XYT stack was gain for 1024 × 1024 pixel resolution and 2 µs/pixel scanning speed for 5 minutes. The XYZ stack was gain for 1024 × 1024 pixel resolution 4 µs/pixel scanning speed for 200 μm. For assay, the velocity, diameter, flux, and line‐scan measurements were designed by 10 μs/pixel scanning rate and 2000 frames in total. Vessel diameters, blood velocity, and flux were calculated with an automated algorithm using MATLAB software.^37^

### 2.10 Western blotting assay

Western blotting analysis was carried out according to protocols as described previously.^38^ In brief, the total brain protein extracts from the cerebral cortex of mice with lysis buffer were prepared for Western blotting. The equivalent amount of protein was separated by 10% acrylamide denaturing gels (SDS‐PAGE) and then transferred to PVDF membrane (Millipore). Membranes were blocked with fat‐free milk for 1 hour and incubated with primary antibodies as following: anti‐β‐Actin (1:5000, Sigma‐Aldrich); anti‐Calcineurin (made by oneself); anti‐spectrin (1:1000, Millipore); anti‐SENP1 (1:2000, Abcam); anti‐FADD (1:500, Santa Cruz); anti‐Fas‐L (1:500, Santa Cruz); anti‐bcl‐2 (1:500, Santa Cruz); anti‐ZO‐1 (1:1000, Invitrogen); and anti‐Occludin (1:1000, Invitrogen) at 4ºC overnight and then incubated with HRP‐conjugated secondary antibodies (1:5000, Life Science). The proteins were visualized by an enhanced chemiluminescence detection system (Amersham Life Science). The density of the bands was quantified with ImageJ software (NIH) and normalized to β‐Actin.

### 2.11 Immunofluorescence assay

Mice were anesthetized and transcardially perfused with PBS immediately followed by 4% paraformaldehyde (PFA) in PBS as previously described.^39^ 50‐μm‐thick sections were prepared by vibratome. The slices were incubated in PBS with 0.01% Triton X‐100 for 15 minutes and in TSA for 1 hour at room temperature. For immunofluorescence, the brain slices were incubated with primary antibodies as following: anti‐SENP1 (1:200, Abcam) and anti‐NG2 (1:200, Abcam) for two night at 4°C. After washing 10 minutes for 3 times, the sections were incubated with Alexa Fluor 488 conjugated anti‐rabbit IgG (Invitrogen) and Alexa Fluor 594 conjugated anti‐mouse IgG (Invitrogen). The nuclei were stained with DAPI (0.5 μg/mL, Vector Laboratories) for 5 minutes. Immunofluorescence confocal microscopy was performed with a confocal laser scanning microscope (Olympus fv3000).

The HBVPs were seeded on coverslips in 24‐well plates for 24 hours and transfected with Senp1 siRNA. 6 hours after HBSS stimulation, the cells were rinsed with PBS and fixed with 4% PFA for 15 minutes, following with permeabilization for 30 minutes. After blocking with 5% BSA for 1 hour, the cells were incubated with anti‐rabbit Cleaved Caspase 3 antibody (1:100, Cell Signaling Test) at 4°C overnight. After washing with PBS, cells were incubated with Alexa Fluor 488 conjugated anti‐rabbit IgG. The nuclei were stained with DAPI for 5 minutes.

### 2.12 Flow cytometric analysis

HBVPs were seeded in 6‐well plates for 24 hours and transfected with Senp1 siRNA. Following 6 hours HBSS treatment, the HBVPs were collected and quantified according to the manufacturer's instructions.^40^ Briefly, the HBVPs were washed with PBS twice and resuspended in binding buffer. Then, the HBVPs were stained with Annexin V/propidium iodide (PI) for 15 minutes at room temperature in the dark and immediately analyzed using a flow cytometer (FACSCalibur). Apoptotic cells were expressed as a percentage of the total number of cells.

### 2.13 Cell coculture and the barrier integrity evaluation

To make a BBB model in vitro with pericytes and endothelial cells, the HBVPs were first seeded on the bottom sides of the Transwell inserts (12‐well plate, 3.0‐μm pore size, Corning, 3402) and directed upside down in the well culture plate. After the HBVPs adhered, the Transwells were inverted and cultured normally for 24 hours. After transfection with Senp1 siRNA for another 24 hours, HBMECs (human brain microvascular endothelial cells) were seeded on the top surface of the insert^41^, ^42^, ^43^ (Figure 6A, upper). 24 hours after coincubation, the cells were treated with HBSS for 6 hours. After glucose deprivation, the medium in the upper and bottom chambers were replaced with DMEM containing 10% FBS and the transendothelial electric resistance (TEER) was detected with a Millicell epithelial‐volt‐ohm meter and chopstick electrodes (Millipore). Subsequently, the electrode was placed in the upper and bottom chambers to measure a TEER value.

To evaluate the permeability of the coculture model after transfection and HBSS treatment, we detect the leakage of Evans blue (EB)‐Albumin across the inserts. Evans blue forms serum protein complex with albumin. 12‐well plates (the bottom chamber) were replaced with 1 mL D‐Hanks buffer (140 mM NaCl, 0.3 mM Na_2_HPO_4_, 0.4 mM KH_2_PO_4_, 5 mM KCl, 4 mM NaHCO_3_) containing 5% bovine serum albumin. In the inserts, the medium was replaced with 0.5 mL buffer with Evans blue (600 μg/mL).^44^, ^45^ 15, 30, and 60 minutes after incubation, 100 μL solution was collected from the bottom chamber and subsequently measured at 620 nm with a microplate reader (infinite F50, TECAN).

To further confirm the role of pericyte‐derived SENP1 on BBB during ischemia, we investigate the expression of TJ proteins with coculture model as shown in Figure 6A (lower).^46^ The HBVPs were seeded on the 6 wells or coverslips in 24‐well plates for 24 hours and transfected with Senp1 siRNA. 24 hours after transfection, the HBMECs was added to HBVPs culture and grown for another 24 hours. 6 hours after HBSS stimulation, the cells were used for Western blotting and immunofluorescence assay, respectively.

### 2.14 Statistics analysis

Statistical analyses were performed using GraphPad Prism 8 (GraphPad Software). Unless otherwise noted, significant differences were determined by either unpaired two‐tailed Student's *t*‐test or one‐way analysis of variance (ANOVA) followed by a post hoc Tukey's Test. Data that do not exhibit a normal/Gaussian distribution were analyzed via a nonparametric equivalent. The results are presented as the mean ± SEM, and *P* < .05 indicated statistical significance.

## 3 RESULTS

### 3.1 Expression profiling identifies *senp1* in pericytes as a potential protective factor in cerebral ischemia

**FIGURE 1:**
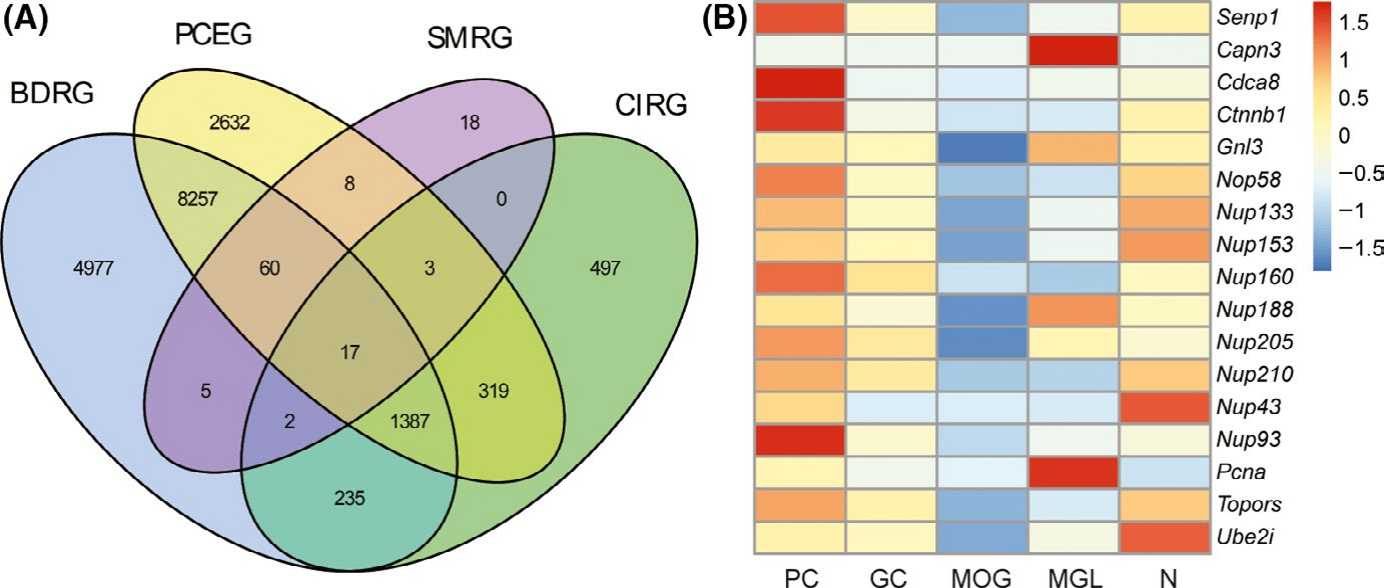
Expression profiling identifies *senp1* in pericytes as an important factor in the cerebral ischemia. A, Venn plot representing the number of intersecting genes. BDRG, brain development‐related genes; PCEG, pericyte‐expressed genes; SMRG, SUMOylation‐related genes; CIRG, cerebral ischemia‐related genes. B, Heatmap of the expression profile of 17 genes in five types of cells. GC, oligodendrocyte precursor cells; MGL, microglia; MOG, myelinating oligodendrocytes; N, neuron; PC, pericytes

In order to identify new molecular mechanisms underlying pericytes protecting brain from cerebral ischemia, we used a database mining strategy with four inclusion criteria: (a) candidate molecules should be important for brain development; (b) distinct expression of candidate molecules in brain pericytes; (c) candidate molecules should be associated with SUMOylation process; and (d) candidate molecules should be associated with brain ischemia. We utilized transcriptional profiling analysis in three database of GEO datasets to obtain intersected DEGs and examined the genes related to SUMOylation from the Human Protein Atlas.^24^, ^25^, ^26^ From this analysis, we identified 17 genes (Figure 1A) and analyzed the expression difference in brain pericytes, GC (oligodendrocyte precursor cells), MOG (myelinating oligodendrocytes), MGL (microglia), and neuron (Figure 1B). *Senp1*, *Cdca8*, *Ctnnb1,* and *Nup93* were highly expressed in pericytes. However, only a significant increase in *senp1* expression could be observed by ischemic preconditioning (IPC) in cerebral ischemia (GSE122107). Therefore, *senp1* displayed significantly different from other genes and matched best to our four postulates. It suggested us that *senp1* in the pericytes may play an important role in cerebral ischemia.

### 3.2 Pericyte‐specific deletion of *senp1* does not affect motor function and learning memory in mice

**FIGURE 2:**
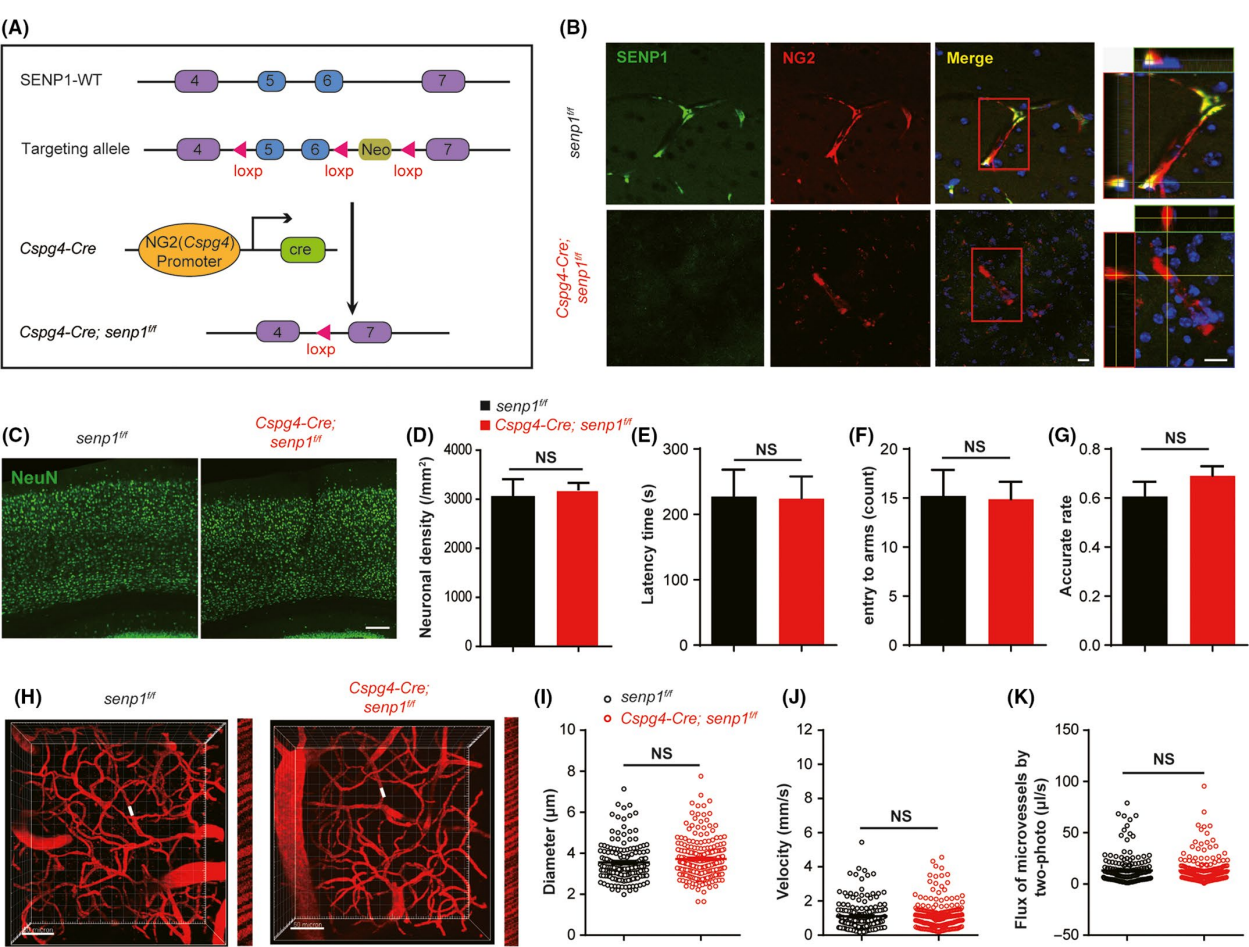
Pericyte‐specific deletion of senp1 does not affect the function of neurons and brain vascular in vivo. A, Schematic diagram of the strategy used to generate conditional pericyte‐specific *senp1* knockout (*Cspg4‐Cre; senp1^f/f^*) mice. B, Representative photographs of fluorescence immunostaining with SENP1 (green) and NG2 (red, a marker for pericytes) in the brain of mice. Scalar bar = 10 μm. The images of fourth column are the orthogonal views of the area in the red box in left photographs. Z = 40 μm. C, Representative immunostaining with NeuN (green, a marker for neurons) and DAPI (blue, a marker for nucleus) in the cortex of brain in mice. Scalar bar = 100 μm. D, Quantitative analysis of neuronal density in the cortex of *senp1^f/f^* mice and *Cspg4‐Cre; senp1^f/f^* mice. n = 3 or 4 mice. E, The latency time to fall during a fixed‐speed rotarod test in *senp1^f/f^* mice and *Cspg4‐Cre; senp1^f/f^* mice. n = 14 or 17 mice. F and G, The count of entering to arms and the accurate rate of Y‐maze test between *senp1^f/f^* mice and *Cspg4‐Cre; senp1^f/f^* mice. n = 14 or 17 mice. H, Mapping the angioarchitecture and line‐scan data of blood flow in capillaries by two‐photon laser scanning microscopy (TPLSM) with Texas‐Dextran labeling in *senp1^f/f^* mice and *Cspg4‐Cre; senp1^f/f^* mice. Z = 200 μm. Scalar bar = 50 μm. The right narrow images represent the blood flow velocity at the white line in the left figures, which is indicated by the slop. I‐K, Quantitative analysis of diameter, velocity, and flux of microvessels in the cerebral cortex of *senp1^f/f^* mice and *Cspg4‐Cre; senp1^f/f^* mice. n = 3 mice. 50‐70 microvessels/mouse. NS: no significant difference. Data were presented as mean ± SEM. See details in Table S1

To investigate the function of *senp1* in pericytes in the brain, we generated the conditional pericyte‐specific *senp1* knockout mice (*Cspg4‐Cre; senp1^f/f^* mice, Figure 2A). SENP1 expression was significantly reduced in pericytes of *Cspg4‐Cre; senp1^f/f^* mice with immunofluorescence suggested the successful deletion of *senp1* in mice (Figure 2B). Next, to evaluate the effect of *senp1* deletion in neurons, we measured NeuN in cortical neurons. The results showed that there was no significant difference on neuronal density between *senp1^f/f^* mice and *Cspg4‐Cre; senp1^f/f^* mice (Figure 2C,D, Figure S4, Table S1). Furthermore, we addressed whether deletion of *senp1* in pericytes caused behavioral changes in mice. In the rotarod test and Y‐maze test, *Cspg4‐Cre; senp1^f/f^* mice showed similar latency time and accurate rate with *senp1^f/f^* mice, indicated that pericytes deletion of *senp1* did not affect the motor function and learning memory (Figure 2E‐G, Figure S4, Table S1).

Then, we observed the brain vascular morphology using TPLSM imaging, including intensity, diameter, velocity, and RBC volume flux of microvessels. We found the density of brain vascular was unchanged, and no significant difference was observed on the diameter, velocity, and flux of microvascular between *senp1^f/f^* mice and *Cspg4‐Cre; senp1^f/f^* mice (Figure 2H‐K, Table S1). All the results demonstrated pericyte‐specific deletion of *senp1* did not affect the function of neurons and brain vascular in mice.

### 3.3 Deletion of *senp1* in pericytes aggravated ischemic injury in mice

**FIGURE 3:**
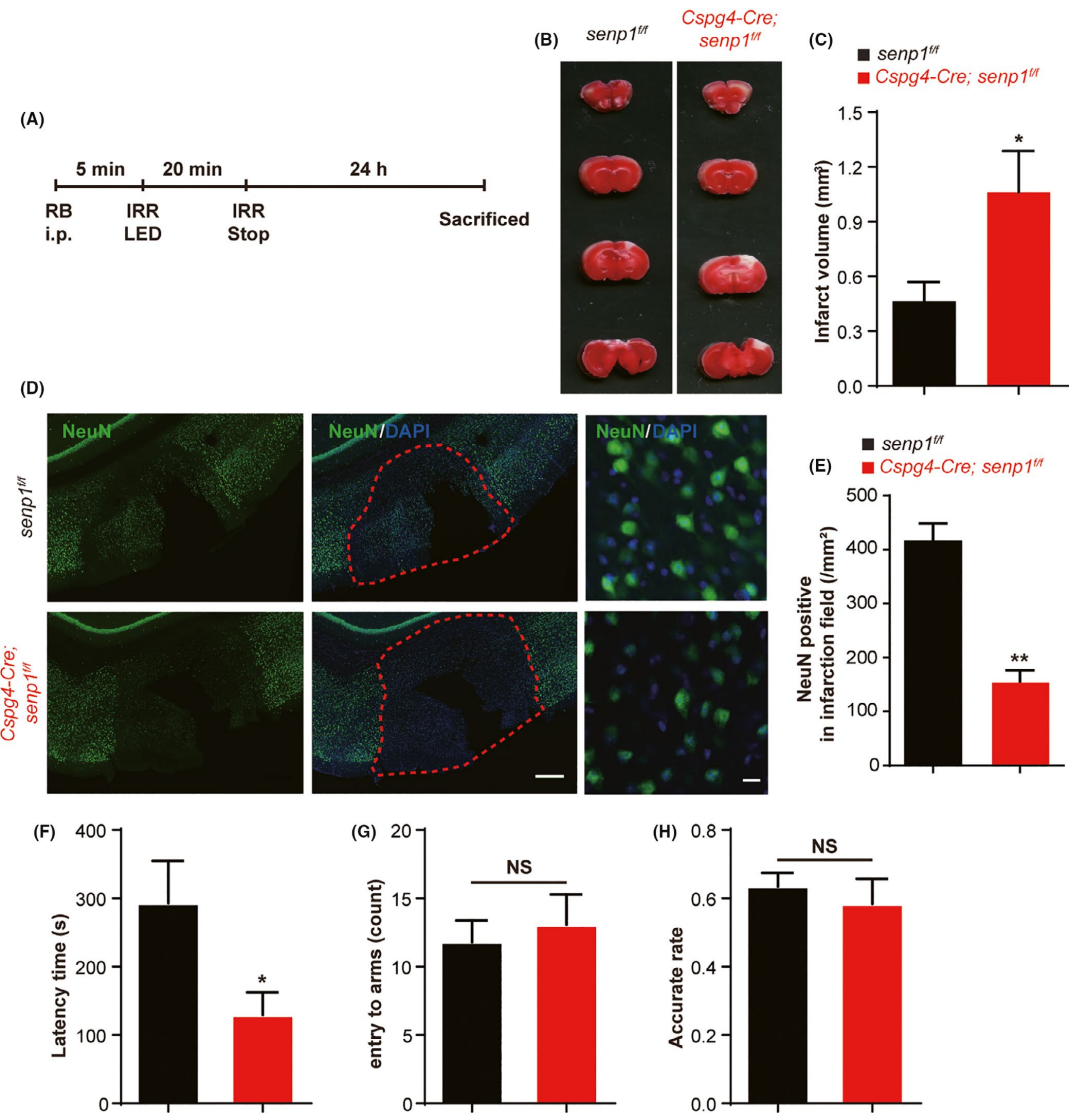
Pericyte‐specific deletion of *senp1* aggravated cerebral ischemic damage and motor function in vivo. A, Experimental protocol for photochemically induced thrombosis (PIT) model induced by Rose Bengal in mice. RB, rose Bengal; IRR, irritate. B, The representative TTC staining of brain slices of *senp1^f/f^* mice and *Cspg4‐Cre; senp1^f/f^* mice 24 h after ischemia. C, Quantitative analysis of cerebral infarct volumes of mice. n = 8 mice. D, Representative immunostaining with NeuN (green) and DAPI (blue) in the cerebral cortex of *senp1^f/f^* mice and *Cspg4‐Cre; senp1^f/f^* mice 24 h after ischemia. Scalar bar = 100 μm. The images in the third column are high magnification for the infarct areas of ischemic mice. Scalar bar = 10 μm. E, Quantitative analysis of NeuN‐positive cells in the infarct areas of *senp1^f/f^* mice and *Cspg4‐Cre; senp1^f/f^* mice 24 h after ischemia. n = 3 mice. F, The latency time to fall during a fixed‐speed rotarod test in mice 24 h after ischemia. n = 14 or 17 mice. G and H, The count of entering to arms and the accurate rate of Y‐maze test in mice 24 h after ischemia. n = 14 or 17 mice. ^*^ *P* < .05, ^**^ *P* < .01 vs *senp1^f/f^*; NS, no significant difference. Data were presented as mean ± SEM. See details in Table S1

Although *senp1* deletion in pericytes has no influence in mice, the effect on brain ischemia is unknown. We use Rose Bengal to generate PIT model for local ischemia to assess the changes in *Cspg4‐Cre; senp1^f/f^* mice, and TTC staining showed the infarction volume of brain was significantly larger in *Cspg4‐Cre; senp1^f/f^* mice than *senp1^f/f^* mice (Figure 3A‐C, Figure S4, Table S1). In addition, the density of neuron decreased significantly in *Cspg4‐Cre; senp1^f/f^* mice in the infarct areas of ipsilateral brain, compared with *senp1^f/f^* mice (Figure 3D,E, Figure S4, Table S1).

Then, we examined whether pericyte‐specific deletion of *senp1* affected motor function and learning memory in ischemic mice. The latency of the rotarod test was markedly decreased in *Cspg4‐Cre; senp1^f/f^* mice after cerebral ischemia (Figure 3F, Figure S4, Table S1). There was no significant difference in the Y‐maze test between *Cspg4‐Cre; senp1^f/f^* mice and *senp1^f/f^* mice, indicated no influence on learning and memory ability (Figure 3G,H, Figure S4, Table S1). These results suggested that SENP1 in pericytes played the protective role in cerebral ischemia of mice.

### 3.4 Deletion of *senp1* in pericytes accelerated thrombosis in mice

**FIGURE 4:**
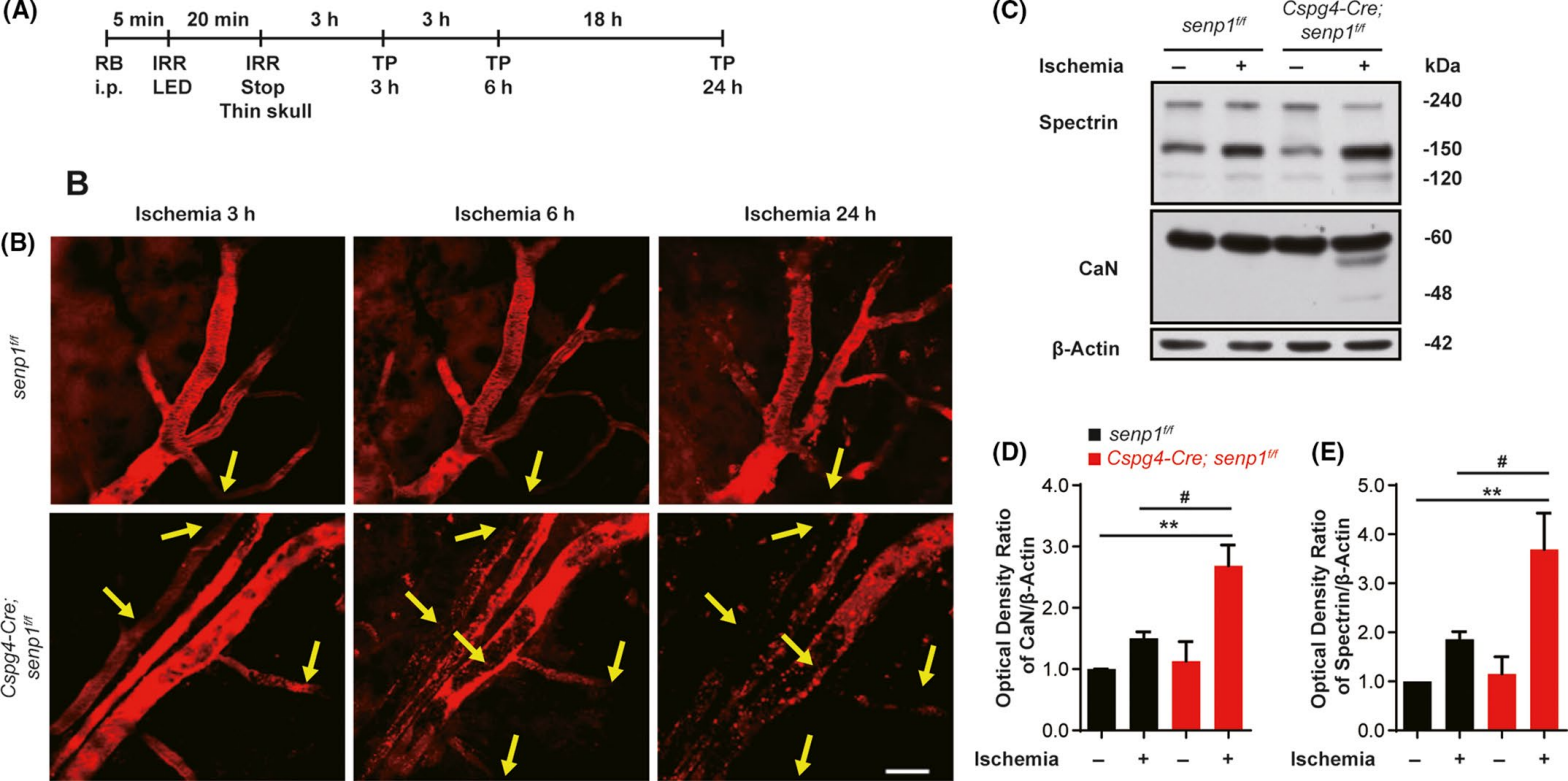
SENP1 deletion in pericytes exacerbated thrombosis in mice after ischemic insults. A, Experimental protocol for PIT model induced by Rose Bengal in mice and TPLSM. RB, rose Bengal; IRR, irritate; TP, Two‐photon imaging. B, Representative two‐photon imaging of vessels 3, 6, and 24 h after ischemia surrounding the infarction of *senp1^f/f^* mice and *Cspg4‐Cre; senp1^f/f^* mice. The mice were administered with Dextran Texas Red (70 kD) intravenously. The yellow arrow indicates the thrombosis. Scalar bar = 30 μm. C, Western blotting assay of CaN and spectrin in the cerebral cortex of *senp1^f/f^* mice and *Cspg4‐Cre; senp1^f/f^* mice 24 h after ischemia. D and E, Quantitative analysis of Western blotting results from C. n = 4 mice. ^**^ *P* < .01 vs *senp1^f/f^*; ^#^ *P* < .05 vs *senp1^f/f^* + Ischemia. Data were presented as mean ± SEM. See details in Table S1

Pericytes are located on the abluminal surface of ECs and provide structural and nutritional support to ECs.^47^ To explain why pericyte‐specific deletion of *senp1* aggravated cerebral ischemic injury, we detect blood flow surrounding the infarct area of ipsilateral brain 3, 6, and 24 hours after ischemia with TPLSM (Figure 4A). The black dots (un‐labeled dye) were found within the vessels which indicating a reduction in blood flow and in some instances completely halted (yellow arrows) after photothrombosis in mice, and the thrombosis was aggravated in *Cspg4‐Cre; senp1^f/f^* mice in a time‐dependent manner (Figure 4B).

However, how *senp1* deletion in pericytes increases brain injury after accelerating thrombosis is unknown. Calcineurin (CaN) is the only calmodulin phosphatase which is regulated by a second messenger, Ca^2+^, especially in those neurons vulnerable to ischemia.^48^, ^49^ The expression of active fragmentation of CaN was increased significantly in the penumbra in *Cspg4‐Cre; senp1^f/f^* mice after cerebral ischemia (Figure 4C,D, Figures S1 and S4, Table S1). Spectrin, which is indispensable for the maintenance of neuronal homeostasis, plays an important role in maintaining plasma membrane integrity and cytoskeletal structure.^50^, ^51^ Similarly, the fragmentation of spectrin accumulated in *Cspg4‐Cre; senp1^f/f^* mice after cerebral ischemia (Figure 4C,E, Figures S1 and S4, Table S1). Collectively, these results indicated that *senp1* deletion in pericytes could aggravate cerebral ischemic insults in mice.

### 3.5 SENP1 knockdown induce apoptosis signaling in pericytes after glucose deprivation

**FIGURE 5:**
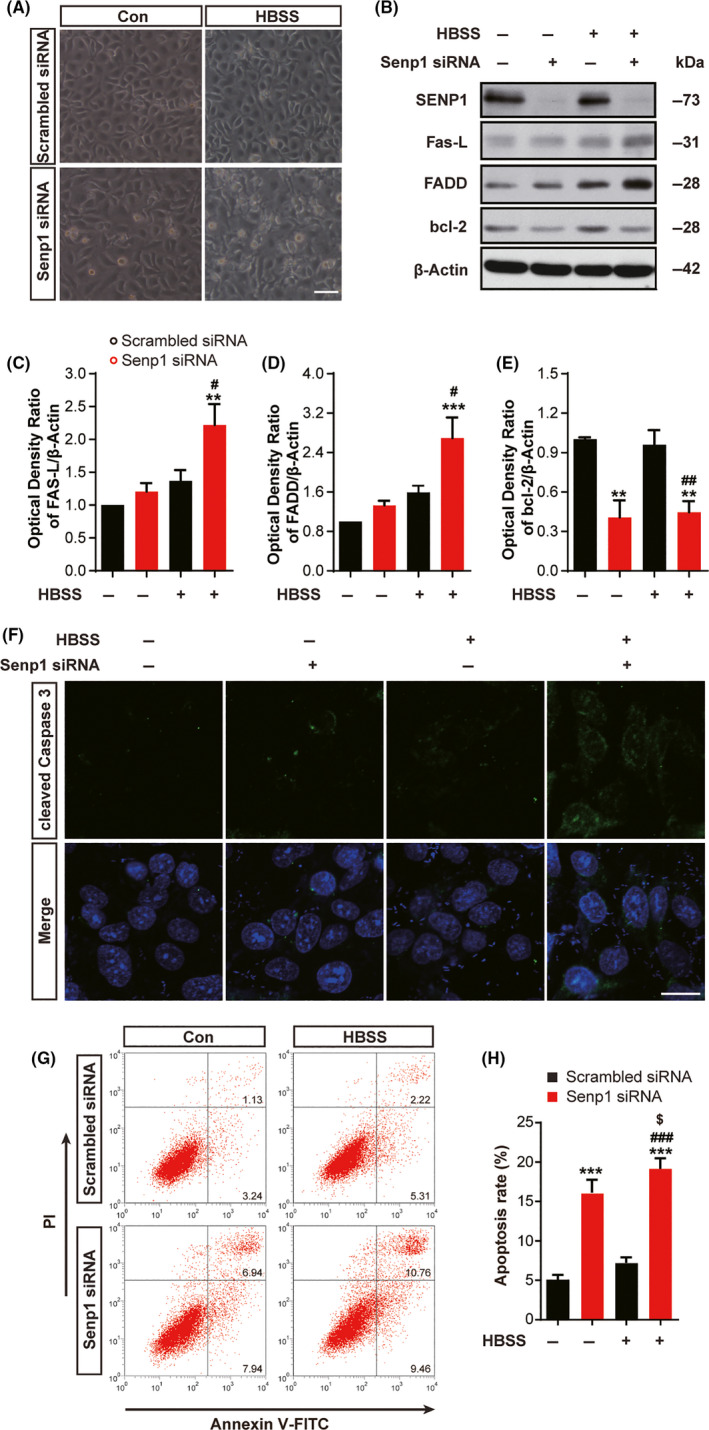
SENP1 knockdown in pericytes activated apoptosis signaling after glucose deprivation. A, The morphology of HBVPs transfected with Senp1 siRNA and treated with HBSS for 6 h. Scalar bar = 100 μm. B, Western blotting assay of SENP1, Fas‐L, FADD, bcl‐2, and β‐Actin in HBVPs after transfection and glucose deprivation. C‐E, Quantitative analysis of Western blotting results from B. n = 5 or 6. F, Representative images of cleaved Caspase 3 (green) and DAPI (blue) immunostaining in HBVPs after transfection and glucose deprivation. Scalar bar = 20 μm. G, Representative images of apoptotic cells with PI and Annexin V‐FITC costaining after transfection and glucose deprivation analyzed by flow cytometry. H, Quantitative analysis of apoptotic cells after transfection and glucose deprivation. Apoptosis rate was the sum of the upper and lower right quadrants of each plot. n = 3. ^**^ *P* < .01, ^***^ *P* < .001 vs Con; ^#^ *P* < .05, ^##^ *P* < .01, ^###^ *P* < .001 vs HBSS‐group; ^$^ *P* < .05 vs Senp1 siRNA‐group. Data were presented as mean ± SEM. See details in Table S1

Next, we sought to elucidate the mechanisms that underlie aggravated cerebral ischemic damage after *senp1* deletion in pericytes. Human brain vascular pericytes (HBVPs) were cultured and transfected with Senp1 siRNA to investigate the function of SENP1 in ischemia‐glucose deprivation model. 6 hours after glucose deprivation, the condition of HBVPs with *senp1* knockdown is worse than that of control cells (Figure 5A). A robust reduction in SENP1 expression indicated the success of *senp1* knockdown in HBVP cells (Figure 5B, Figure S2). Previously, Zhang et al reported that SENP1 played an important role against apoptosis of cortical neurons in response to I/R.^23^ We wondered if *senp1* knockdown in pericytes activated apoptosis to exacerbate the cerebral ischemic damage. To investigate the role of SENP1 on cell apoptosis, we focus on the expression of the apoptosis‐related proteins, such as Fas‐L, bcl‐2, and cleaved caspase 3 during brain ischemia. SENP1 knockdown could activate the expression of Fas‐L and Fas‐associated death domain (FADD) proteins significantly 6 hours after HBSS stimulation, compared with control cells and HBSS‐treated cells (Figure 5B‐D, Figure S2 and S4, Table S1). SENP1 knockdown down‐regulated bcl‐2 in HBVPs treated with or without HBSS (Figure 5B,E, Figures S2 and S4, Table S1). Furthermore, cleaved Caspase 3 immunofluorescence significantly increased in HBVPs after SENP1 knockdown and HBSS treatment (Figure 5F).

To further confirm whether SENP1 knockdown could exacerbate apoptosis after HBSS stimulation, we evaluated Annexin V/PI expression in HBVPs by flow cytometric analysis. The apoptotic rate was increased to 20.22% after SENP1 knockdown and HBSS treatment, compared with 4.37% in the control group (Figure 5G,H, Figure S4, Table S1). All these results suggested that SENP1 knockdown in pericytes could activate the apoptosis pathway after glucose deprivation.

### 3.6 SENP1 knockdown in pericytes result in BBB disruption in vitro

**FIGURE 6:**
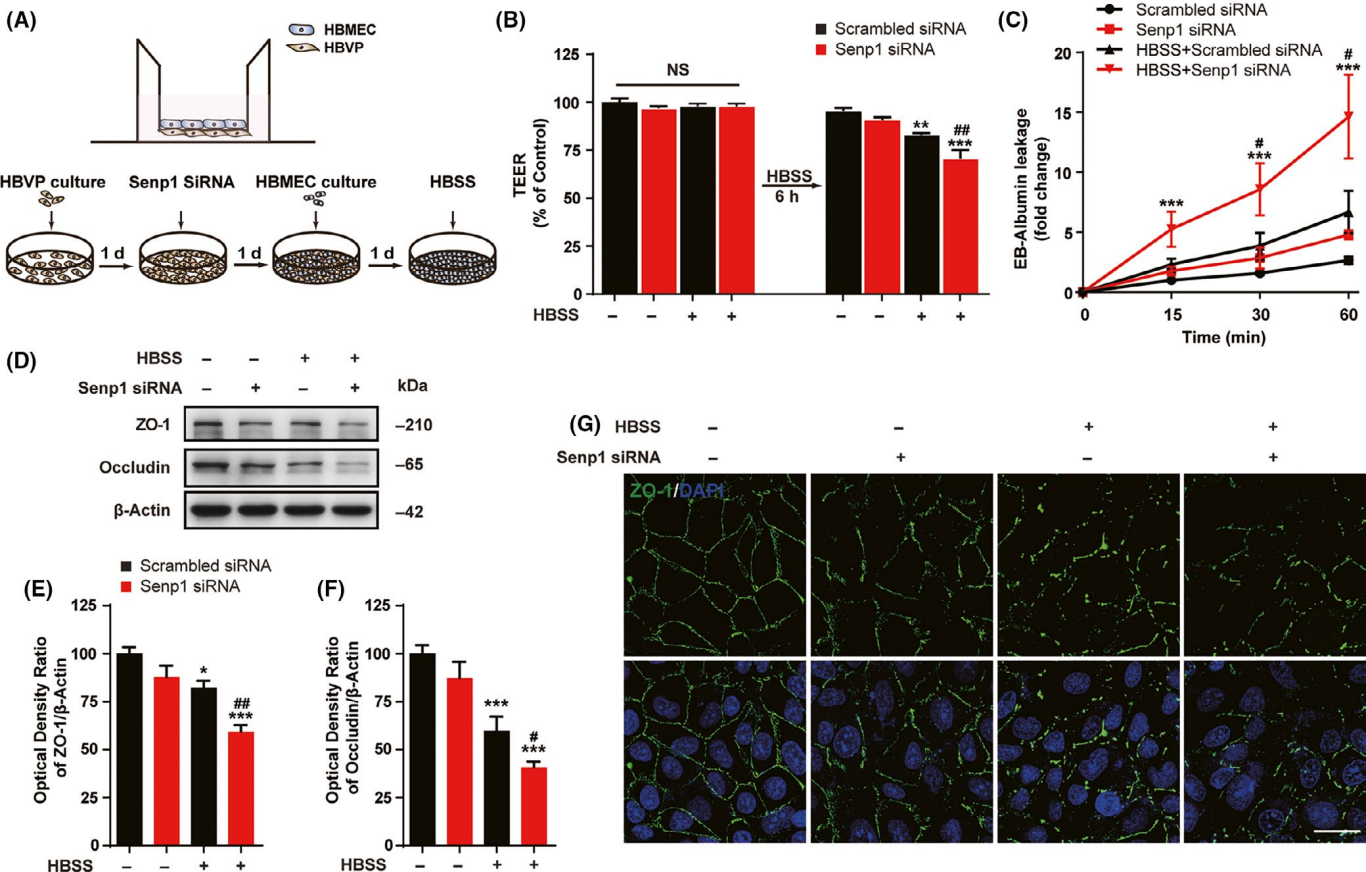
SENP1 knockdown in pericytes could disrupt BBB in vitro. A, Schematic representation of cocultures of HBVPs and HBMECs with Transwell inserts (upper) or in culture dishes (lower). Upper: HBVPs were first plated on the bottom side of the insert and 6 h later, the inserts were turned upside down and cultured for 24 h. The HBVPs were transfected with Senp1 siRNA for 24 h, and HBMECs were seeded on the top surface of the inserts for another 24 h. On the fourth day, the cocultures were treated with HBSS for glucose deprivation for 6 h. The Transwell inserts were used for TEER and the leakage of EB‐Albumin measurements. Lower: Cocultures in 6‐well plates and 24‐well plates with the same time processing of HBVPs and HBMECs were used for Western blotting and immunofluorescence assay, respectively. B, Transendothelial electrical resistance (TEER) measurement from cocultures before and after glucose deprivation. n = 8. NS, no significant difference. ^**^ *P* < .01, ^***^ *P* < .001 vs Con; ^##^ *P* < .01 vs HBSS‐group. C, BBB permeability was evaluated by Evans blue (EB) leakage in the bottom chamber at 15, 30, and 60 min after HBSS treatment in vitro. n = 8. ^***^ *P* < .001 vs Con; ^#^ *P* < .05 vs HBSS‐group at the same time point. D, Western blotting assay of TJ proteins ZO‐1 and Occludin in cocultures after transfection and glucose deprivation. E and F, Quantitative analysis of Western blotting results from D. n = 6. ^*^ *P* < .05, ^***^ *P* < .001 vs Con; ^#^ *P* < .05, ^##^ *P* < .01 vs HBSS‐group. G, Representative images of ZO‐1 (green) and DAPI (blue) immunostaining in cocultures after transfection and glucose deprivation. Scalar bar = 20 μm. Data were presented as mean ± SEM. See details in Table S1

To further explore the role of pericyte‐derived SENP1 in ischemic damage, we coculture pericytes and endothelial cells to make a BBB model in vitro.^41^, ^42^, ^43^ We then evaluated the barrier integrity by measuring the TEER and the permeability to Evans blue with Transwell inserts. As results shown in Figure 6B, there was no significant difference in TEER before HBSS stimulation in cocultures treated with or without Senp1 siRNA. However, SENP1 knockdown in the HBVPs decreased the TEER significantly after HBSS treatment (Figure 6B, Figure S4, Table S1). Evans blue leakage revealed that SENP1 knockdown in pericytes increased the permeability of the HBVPs and HBMECs coculture model (Figure 6C, Figure S4, Table S1). Furthermore, SENP1 knockdown in pericytes decreased the expression of TJ proteins, including ZO‐1 and Occludin, after glucose deprivation significantly (Figure 6D‐F, Figures S3 and S4, Table S1). Immunofluorescence staining also demonstrated that ZO‐1 expression decreased markedly after SENP1 knockdown and HBSS treatment in the coculture model, which suggested the breakdown of BBB (Figure 6G). All these results indicated that SENP1 knockdown in pericytes could increase the permeability of BBB and disrupt the BBB in a coculture model.

## 4 DISCUSSION

Increasing researchers implicated the function of SUMOylation in regulation of apoptosis and protein stability.^52^ However, whether and how SUMOylation in pericytes is involved in cerebral ischemic injury remains unknown. In present study, we first found that cerebral ischemia could cause more severe brain damage in *Cspg4‐Cre; senp1^f/f^* mice than control mice, including infarct volume, motor deficits, NUV injury, and vascular thrombosis. In addition, SENP1 knockdown in HVBPs promoted apoptosis signaling and increased the permeability of BBB in vitro after glucose deprivation.

SUMOylation is a common posttranslational modification targeting on various proteins of cells after ischemia.^53^, ^54^ The exact role of SUMOylation is still discussed controversially. Previous studies have reported that SUMO‐conjugated proteins are increased after ischemia, which are believed to play major effects on the cell fate after stroke.^18^, ^55^ SUMO conjugations were increased both in the hippocampus and striatum in rats treated with transient middle cerebral ischemia.^18^ Furthermore, Lee et.al demonstrated that elevated SUMO conjugation level protected neurons from oxygen and glucose deprivation (OGD) treatment and contributed to ischemic tolerance.^56^ SUMO knockdown mice exhibited severe functional outcomes significantly compared with wild‐type mice after transient ischemia, which further supporting the protective role of SUMOylation in ischemia.^57^

It is very interesting that not only the SUMOylation level but also SENP1 expression were enhanced in cultured neurons after OGD treatment, suggesting that both SUMOylation and deSUMOylation may involve in the neuronal response to OGD.^58^ Some studies also noticed that no significant changes in SUMO1‐conjugated proteins after transient ischemia both in vivo and in vitro.^59^, ^60^ Therefore, as many researchers indicated the effects of SUMOylation was depending on the type of protein and the time occurring.^23^ A global change of SUMOylation in tissue does not show direct evidence whether the modification would be beneficial or detrimental in ischemia process. It is difficult to clarify the precise relationship of SUMOylation and cerebral ischemia in specific cell types. In that condition, we focus on the enzymes regulating the SUMOylation process instead of SUMOylation to consider the potential target for brain protection. Among those enzymes, SENP1 had a broad specificity for SUMO‐1 and SUMO‐2/3 and involved in both their maturation and deconjugation.^20^ In addition, SENP1 expression is up‐regulated in ischemia condition, and further supporting the importance of SENP1 in ischemia.^23^, ^61^

As the main component of the NVU, pericytes provide support to other NVU members and help to maintain normal functions of the NVU.^29^, ^62^ In addition, pericytes can control blood flow in CNS microvessels.^47^, ^63^ Pericyte loss or dysfunction is involved in BBB dysfunction and contributing to neurodegeneration, including stroke.^12^, ^64^ Thus, information about molecular on how pericytes affect cerebral ischemia pathologies may lead to future therapies for stroke. We used a database mining strategy to identify the molecular underlying the protection of pericytes from cerebral and got *senp1* again successfully (Figure 1). It suggested us that pericyte‐derived SENP1 may play an important role in the cerebral ischemia. By using conditional knockout of *senp1*, we found SENP1 in pericytes are obligately linked and can, in fact, be associated with pathological process of cerebral ischemia. The lack of SENP1 in pericytes promoted the ischemic injury in mice, with increased neuronal loss and vascular thrombosis (Figures 3 and 4). We first reported SENP1 in pericytes played a protective role in cerebral ischemia with *Cspg4‐Cre; senp1^f/f^* mice. In this study, photothrombosis induces a small area of injury, mainly in the cortex, which could cause minor sensorimotor deficits. Therefore, this might be the reasons that no significant difference on the count of entering to arms and the accurate rate of Y‐maze test in mice 24 hours after ischemia between *Cspg4‐Cre; senp1^f/f^* mice and *senp1^f/f^* mice.

As we known, apoptosis contributes to a significant proportion of neuronal death following brain ischemia. SENP1 in neurons could protect against apoptotic cell death, which may mediate the regulation of mitochondrial abnormities.^23^, ^65^ Importantly, many target proteins for SUMO are transcription factors and other nuclear proteins which modulate gene expression.^66^ For example, SENP1 could deconjugate SUMOylated HIF‐1α and inhibit the degradation of HIF‐1, thereby promoting transcription of HIF‐1α‐dependent genes.^52^ SENP1 deficit impairs the HIF‐1α signaling and aggravates ischemic damage in myocardial cells.^61^ In addition, peroxynitrite (ONOO^‐^) could induce p53 SUMOylation, which subsequently cause p53 nuclear export and apoptosis in vitro.^67^ Consistent with these reports, we found that SENP1 knockdown could result in increased apoptosis significantly in HBVPs after stimulation in the present study (Figure 5). Thus, we concluded that the aggravated apoptosis (Fas mediated apoptosis pathway) induced by SENP1 knockdown may at least in part mediate the ischemic injury in mice.

**FIGURE 7:**
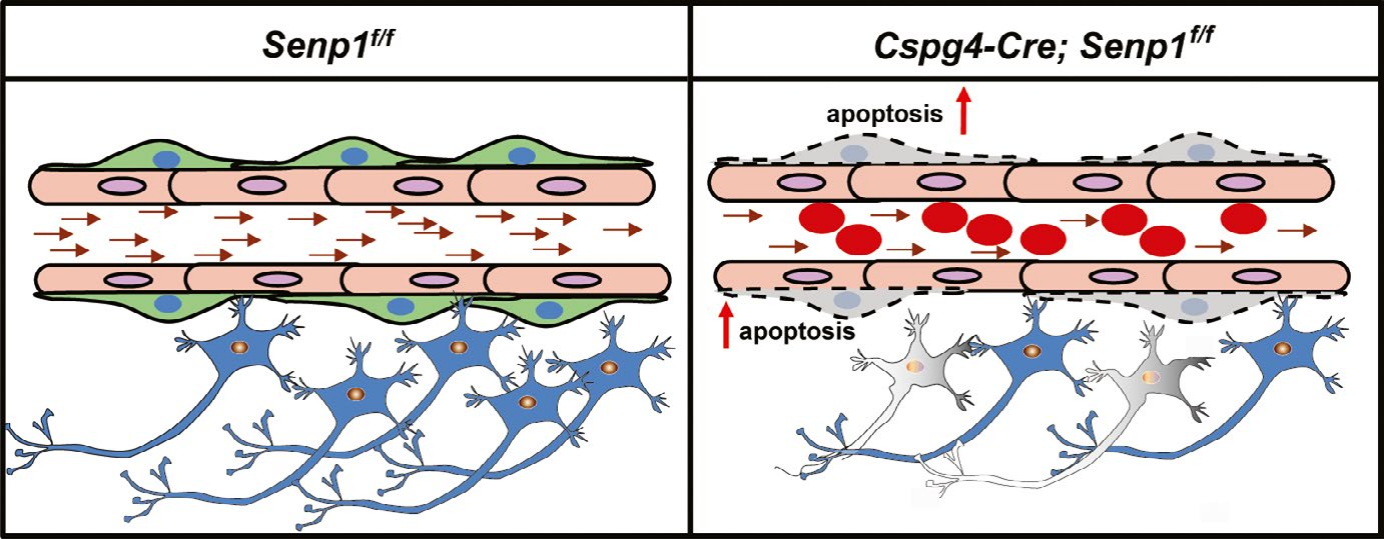
A scheme for the proposed mechanisms underlying the protective role of SENP1 in cerebral ischemia. During ischemic condition, thromboembolic occlusion of the blood vascular occurs, leading to the death of neurons. However, pericyte‐specific deletion of *senp1* could activate the apoptosis signaling in the pericytes in cerebral ischemia, which induced exacerbated thrombosis and worse BBB breakdown, consequently aggravated brain ischemic damage in mice. In conclusion, SENP1 in pericytes plays a protective role in cerebral ischemia

Regarded as a major component of the BBB, pericytes is important for BBB functions and might result in stroke pathogenesis via regulating BBB integrity.^68^ We next examined barrier integrity with coculture model and found that SENP1 knockdown in pericytes could increase the TEER and permeability of the barrier, and decrease the TJ proteins expression. Although we have focused on critical role of pericyte SENP1 signaling during brain ischemia, other events may be equally important in the modulation of pericyte function. Pericytes may also contribute to postinjury brain recovery by releasing pro‐regenerative molecules, such as brain‐derived neurotrophic factor (BDNF),^69^ and regulating the release of cytokines from endothelial cells.^70^ Furthermore, a recent study demonstrated that pericytes can be induced into cholinergic neurons mediating by Myt1l, which suggested the pluripotent properties of pericytes.^71^ Ursula I. Tuor et al^72^ ever reported that diffuse mild ischemic injury surrounding a small photothrombotic lesion could model clinical minor strokes with a penumbra. Notably, microglia/macrophages polarization dynamics in the penumbra of photothrombotic stroke model is interesting^73^, ^74^ and warrants further investigation. Above all, we hypothesized that SENP1 in pericytes may initiate apoptosis signaling and damage pericytes function, which lead to decreased blood flow and vascular thrombosis, consequently resulting in aggravated ischemic insults (Figure 7). Our results strongly suggested the importance of pericyte‐derived SENP1 in cerebral ischemic injury. The increased understanding of the regulation and function of the posttranslational modifier SUMO may provide new targets for therapeutic intervention in neurovascular disorders.

## 5 CONCLUSIONS

SENP1, a SUMO‐specific proteases 1, plays a protective role in pericytes after cerebral ischemia in mice. The pericyte‐specific deletion of *senp1* aggravated the infarct size and motor deficit following focal brain ischemia by activated apoptosis signaling and increased permeability of BBB. Pericytes‐derived SENP1 may be a potential target for protecting brain from ischemic stroke in future studies.

## CONFLICT OF INTEREST

The authors declare no conflict of interest.

## Supporting information

Fig S1‐S4

Fig S1

Fig S2

Fig S3

Fig S4

Table S1
