# Supplementary material for: Role of pericyte‐derived SENP1 in neuronal injury after brain ischemia
Source: CNS Neurosci Ther. 2020 Jun 4;26(8):815–28. doi: 10.1111/cns.13398 (PMC7366739; doi:10.1111/cns.13398)
Supplement: Supplementary file 1 — Fig S1‐S4 [file CNS-26-815-s001.pdf]

Supplementary Figures

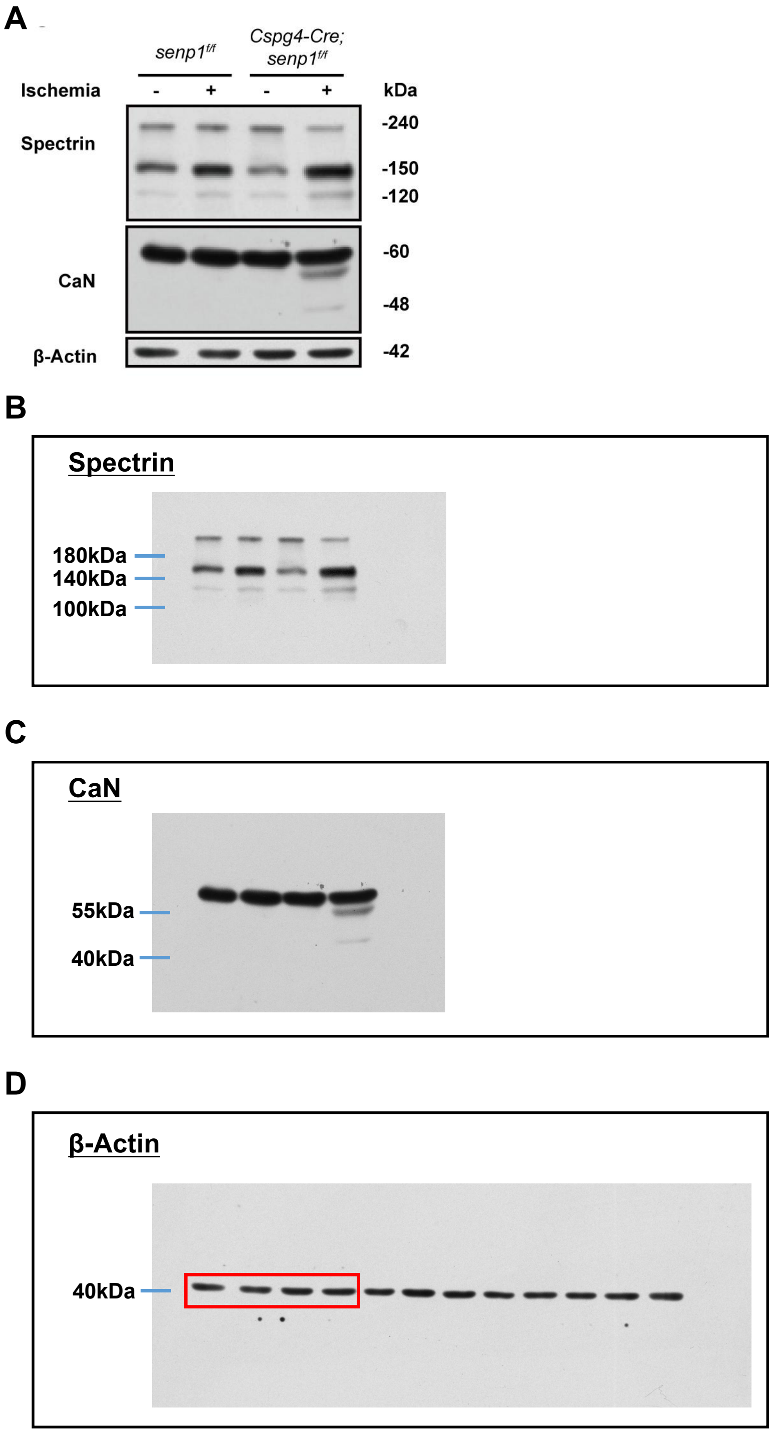

Figure S1 Full unedited gel/blot for Figure 4-C.

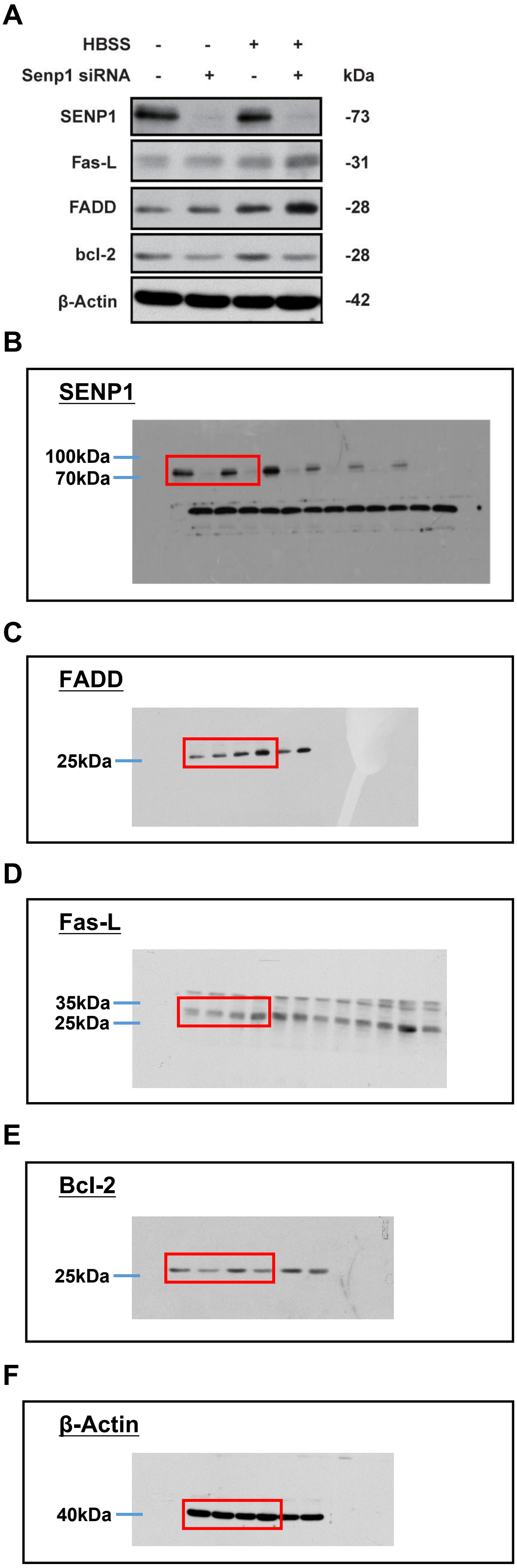

Figure S2 Full unedited gel/blot for Figure 5-B.

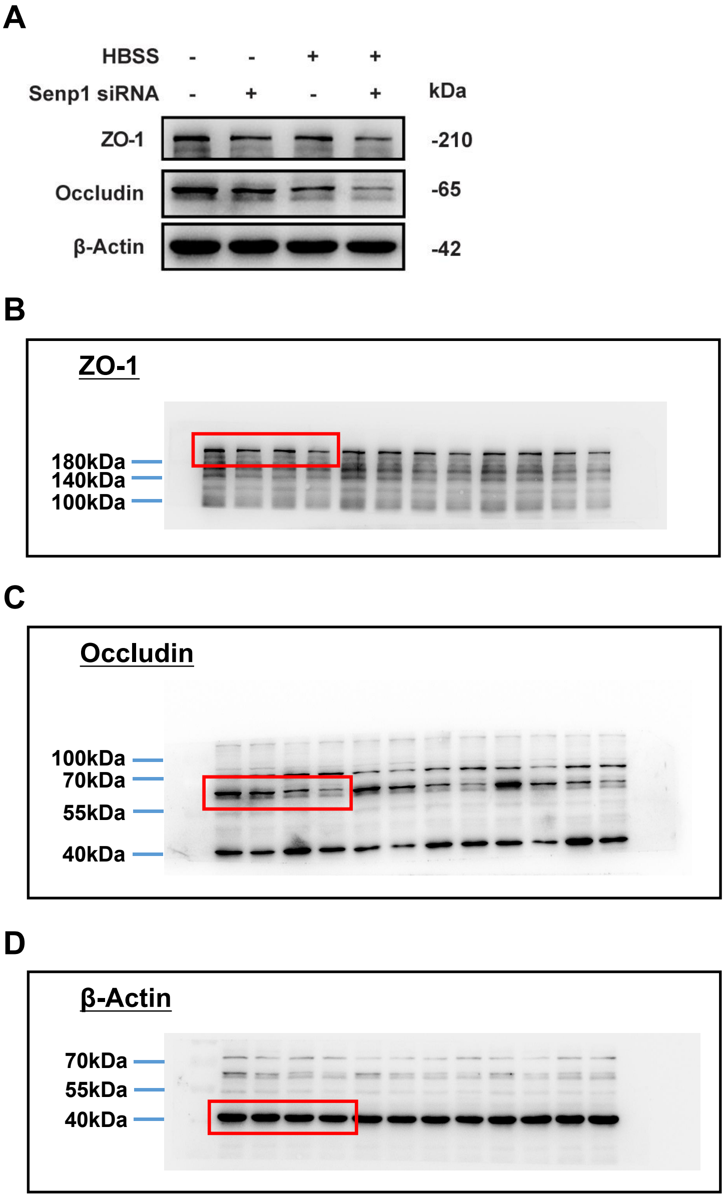

Figure S3 Full unedited gel/blot for Figure 6-D.

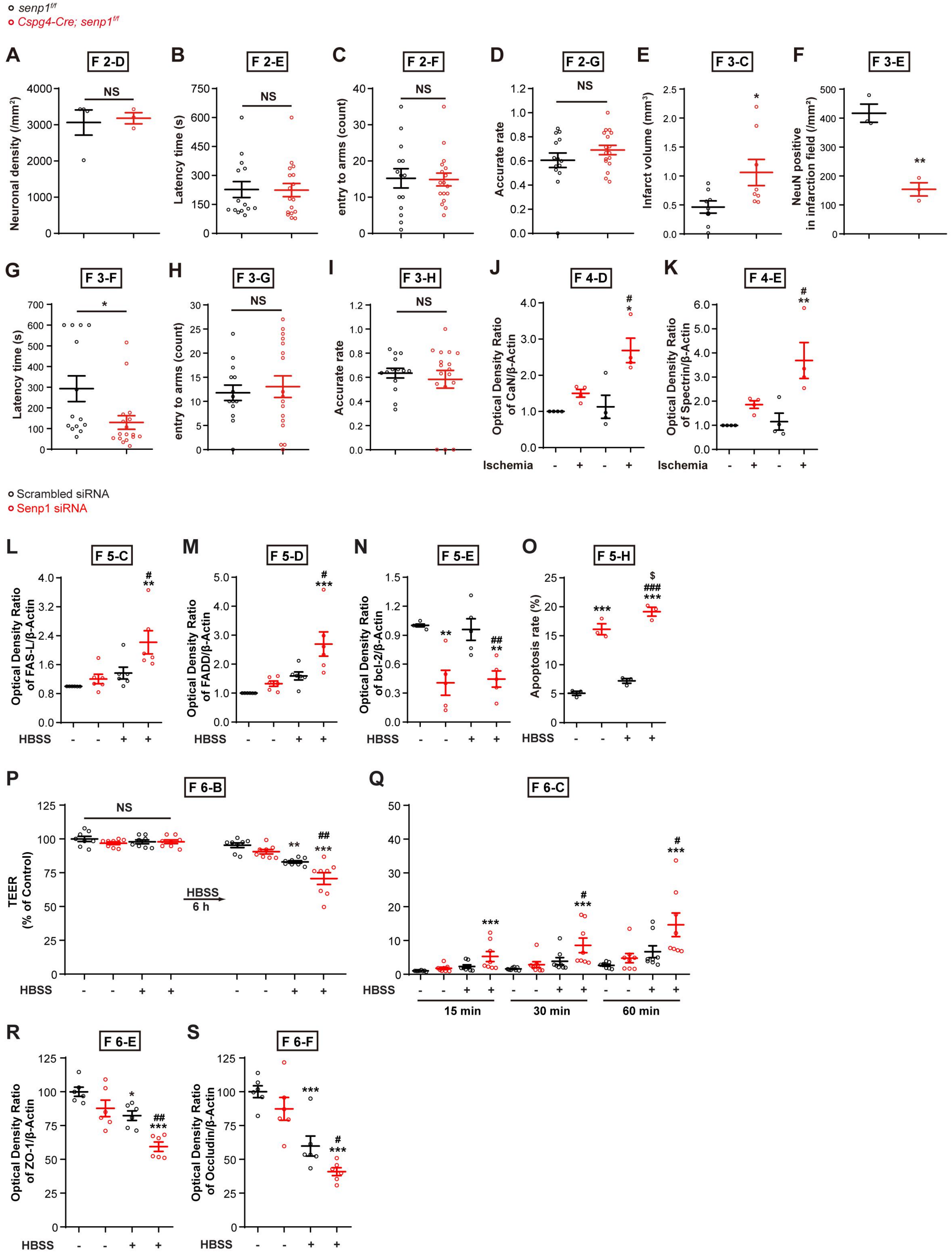

Figure S4 The dot plots for bar plots presented in Figure 2-6.
