# Supplementary figures and images for: Role of pericyte‐derived SENP1 in neuronal injury after brain ischemia
Source: CNS Neurosci Ther. 2020 Jun 4;26(8):815–28. doi: 10.1111/cns.13398 (PMC7366739; doi:10.1111/cns.13398)

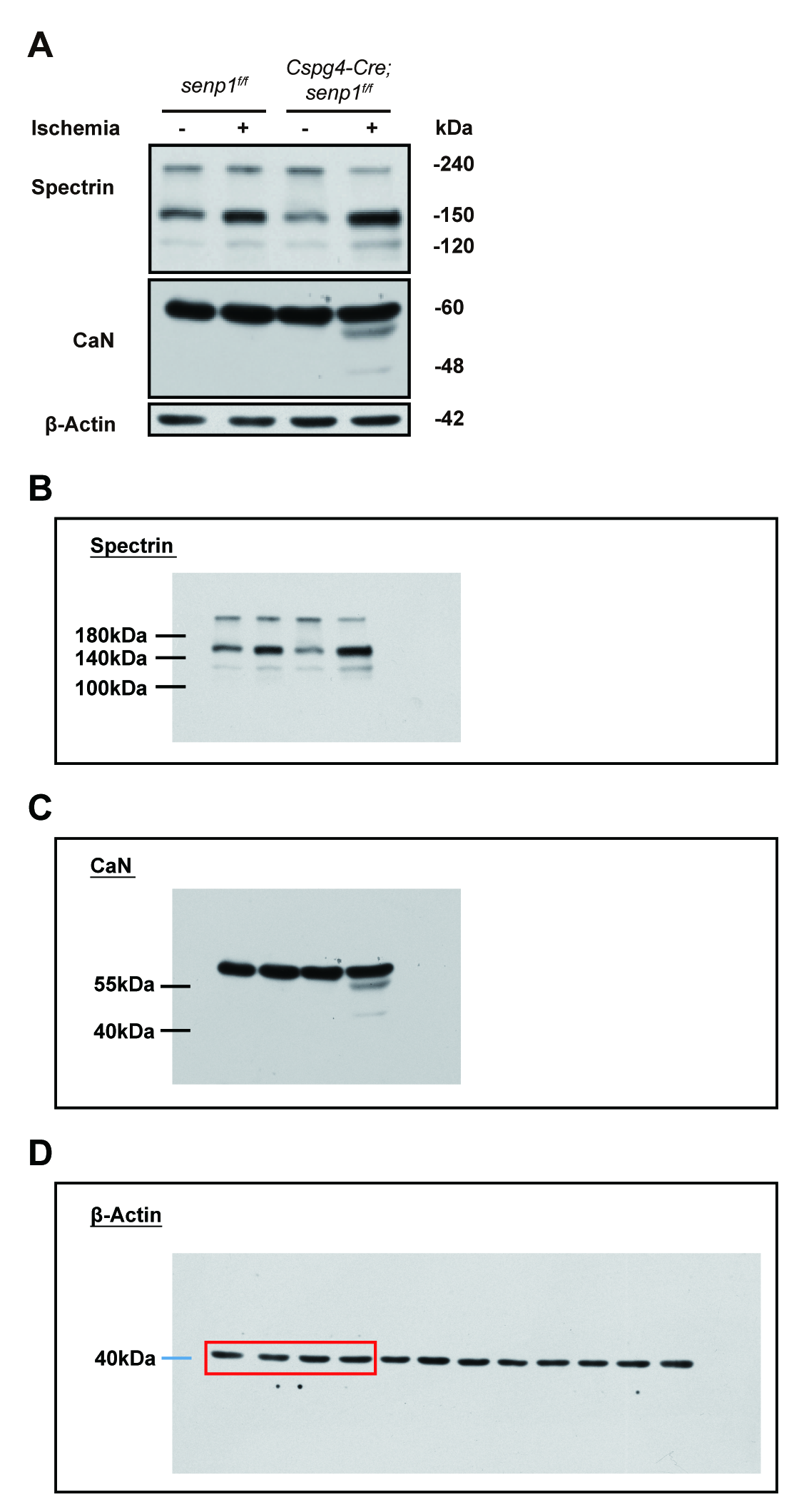

Supplement: Supplementary file 2 — Fig S1 [file CNS-26-815-s002.tif]

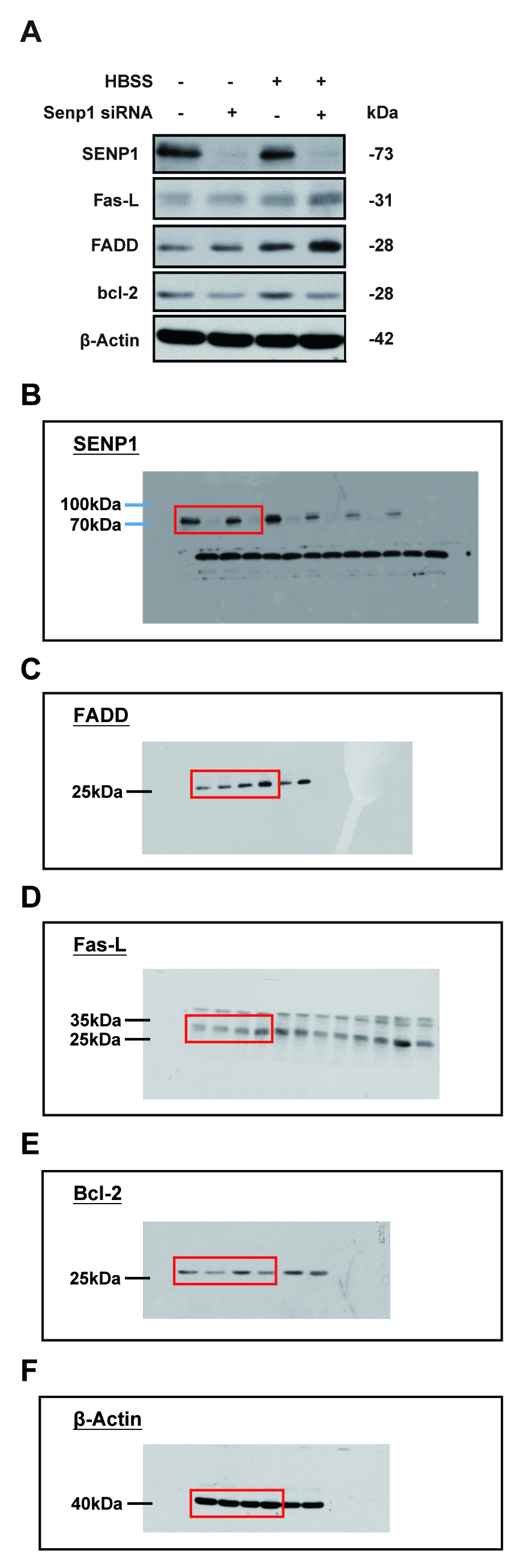

Supplement: Supplementary file 3 — Fig S2 [file CNS-26-815-s003.tif]

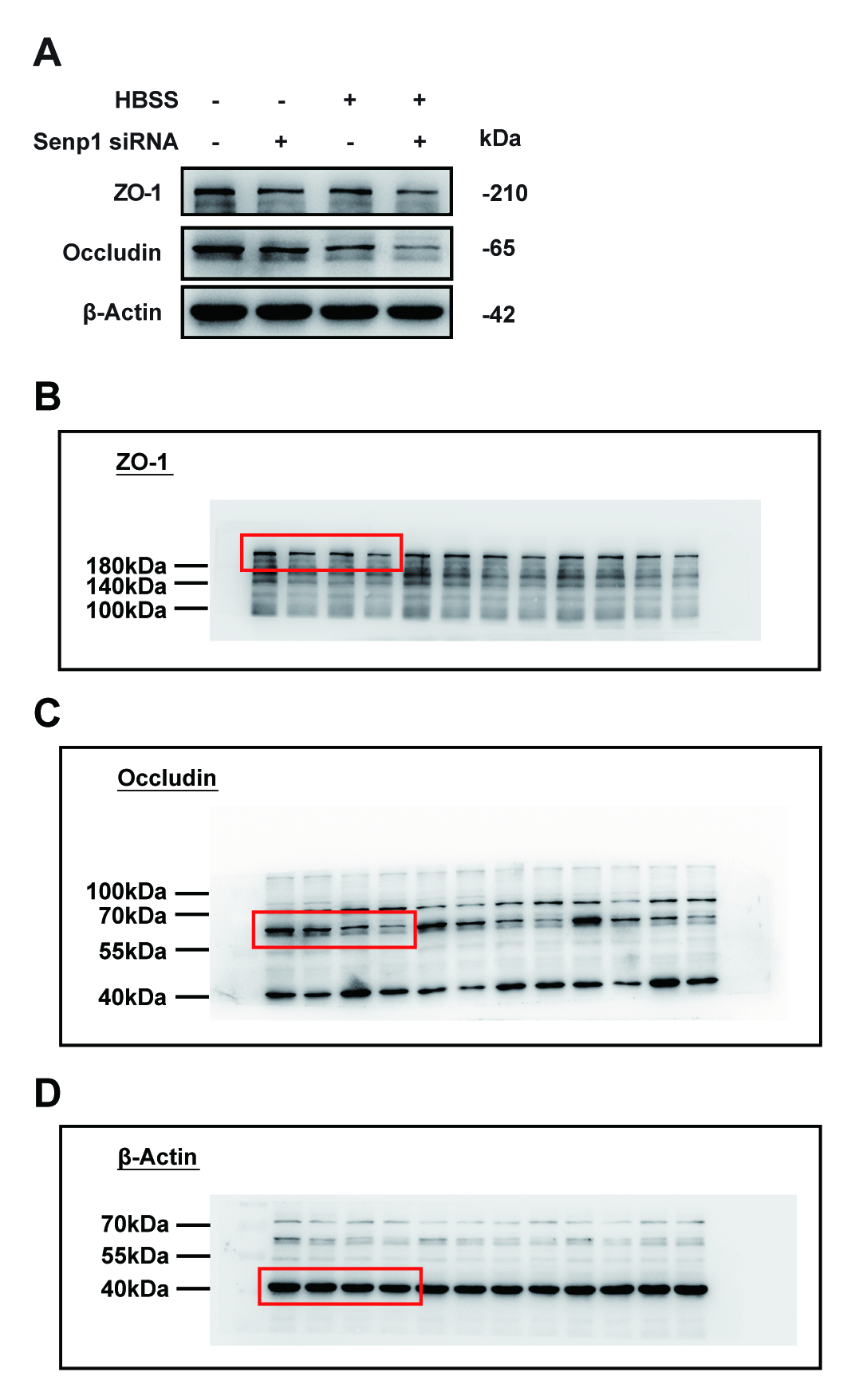

Supplement: Supplementary file 4 — Fig S3 [file CNS-26-815-s004.tif]

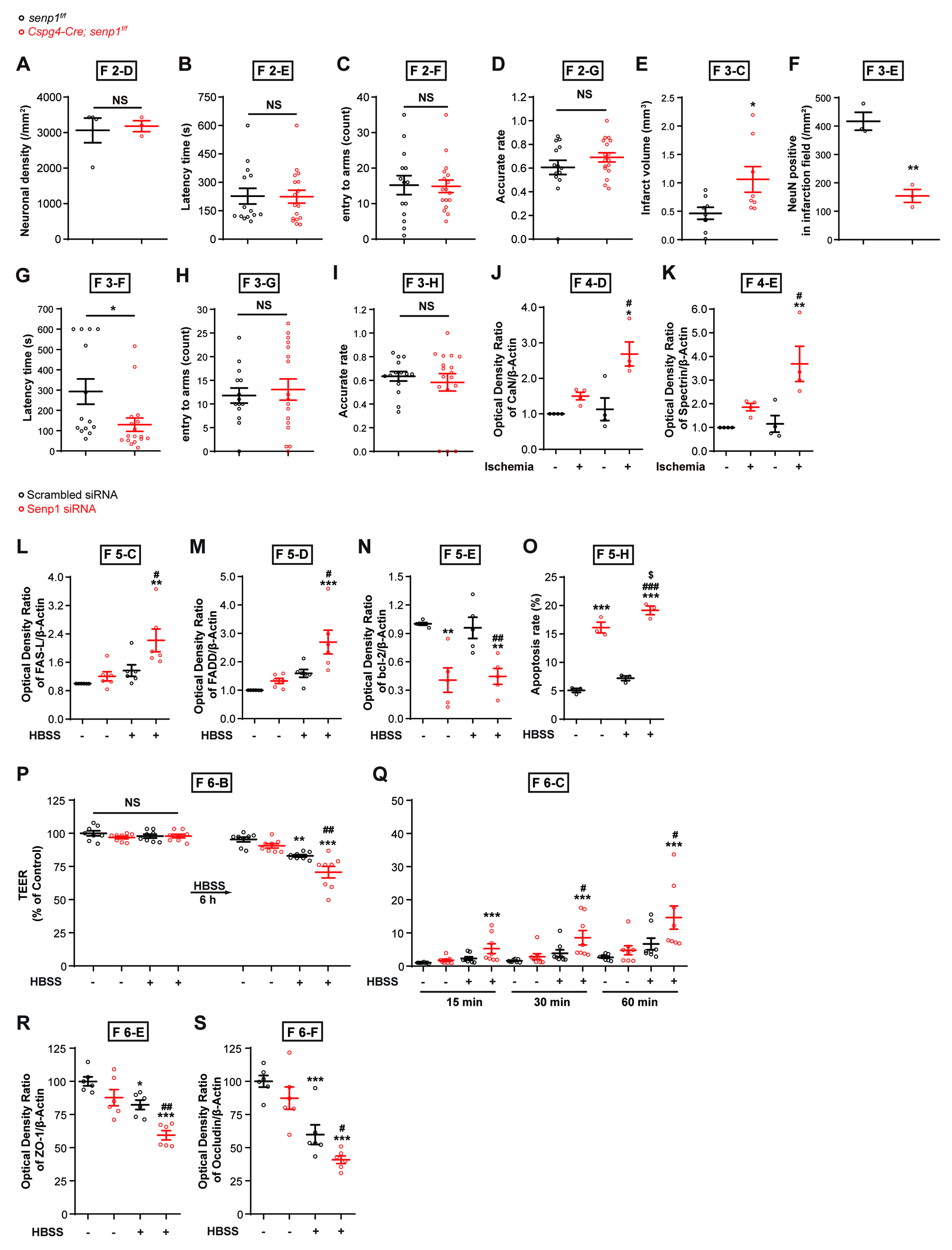

Supplement: Supplementary file 5 — Fig S4 [file CNS-26-815-s005.tif]
